# Tris[tri­phenyl­anti­mony(V)]hexa(μ-oxido)­tellurium(VI): a mol­ecular complex with six Te—O—Sb bridges

**DOI:** 10.1107/S2056989021011294

**Published:** 2021-11-04

**Authors:** Kostiantyn V. Domasevitch, Ganna A. Senchyk

**Affiliations:** a Inorganic Chemistry Department, National Taras Shevchenko University of Kyiv, Volodymyrska Str. 64/13, 01601 Kyiv, Ukraine

**Keywords:** crystal structure, triorgano­anti­mony(V), hexa­oxido­tellurate(VI), poly­oxo­anions, oxide clusters, Hirshfeld surface

## Abstract

In the structure of the title compound [systematic name hexa-μ-oxido-1:2κ^4^ *O*:*O*;1:3κ^4^ *O*:*O*;1:4κ^4^ *O*:*O*-nona­phenyl-2κ^3^ *C*,3κ^3^ *C*,4κ^3^ *C*-tri­anti­mony(V)tel­lur­ium(VI)], [Sb_3_Te(C_6_H_5_)_9_O_6_], the hexa­oxidotellurate(VI) ion is coordinated to three Sb^V^ ions *via* pairs of *cis*-positioned O atoms to form a discrete mol­ecular unit. The Te^VI^ and Sb^V^ central ions exhibit distorted octa­hedral [TeO_6_] and distorted trigonal–bipyramidal [SbC_3_O_2_] coordination geometries, respectively. The linking of these polyhedra, by sharing the dioxide edges, results in the Te-based octa­hedron having a *mer*-configuration. The packing of the mol­ecules is dominated by C—H⋯O hydrogen bonding and weak dispersion forces, with a minor contribution from C—H⋯π bonds and π–π stacking inter­actions. According to the Hirshfeld surface analysis, the contributions of the H⋯H, H⋯C/C⋯H and H⋯O/O⋯H contacts are 58.0, 32.6 and 7.8%, respectively. The title structure provides a model for the bonding of triorgano­anti­mony dications to octa­hedral oxoanions, and the observed doubly bridged motifs, Te(μ-O)_2_Sb, may find application in the functionalization of polyoxometalate species.

## Abstract 2

The structure of (C_18_H_15_Sb)_3_TeO_6_, contains a [TeO_6_] octa­hedral unit linked to three trigonal–bipyramidal [SbC_3_O_2_] units *via* pairs of bridging O atoms to form a discrete mol­ecular unit. The packing of the units is dominated by C—H⋯O hydrogen bonding and weak dispersion forces, with a minor contribution from C—H⋯π bonds and π–π stacking inter­actions.

## Chemical context

Organo­anti­mony(V) species readily form covalent derivatives with a range of organic and inorganic oxo-ions and these can be used in the construction of metal–oxide clusters (Nicholson *et al.*, 2011 ▸). Unlike the series of mol­ecular fivefold-coordinated tetra­phenyl­anti­mony(V) compounds, which easily dissociate in solution to yield tetra­phenyl­stibonium cations, [Ph_4_Sb]^+^ (Domasevitch *et al.*, 2000 ▸), the derivatives of tri­phenyl­anti­mony(V) are much more chemically robust and they are well suited for the preparation of covalent oxide materials. The inter­actions between the Ph_3_Sb^2+^ cations and oxoanions are particularly important as they potentially control the assembly of these units into either discrete oxo-clusters or polymers. For example, one-dimensional covalent chains of oxo-bridged Ph_3_Sb^2+^ moieties were identified as a possible motif for amorphous [Ph_3_SbO]_*n*_ formation (Carmalt *et al.*, 1996 ▸). In addition, there are a few complexes known in which singly charged oxoanions form mol­ecular five-coordinate structures with terminal [ReO_4_]^−^ (Wirringa *et al.*, 1992 ▸) or [PhSO_3_]^−^ (Rüther *et al.*, 1986 ▸) groups or bridging [Ph_2_PO_2_]^−^ groups (Srungavruksham & Baskar, 2013 ▸), while insoluble derivatives with tetra­hedral dianions, such as SO_4_^2−^, SeO_4_^2−^ and CrO_4_^2−^, are likely to be polymeric (Goel *et al.*, 1969 ▸).

At the same time, Ph_3_Sb^2+^ units may coordinate to the O atoms of octa­hedral oxoanion species to form discrete mol­ecules: one can anti­cipate using Ph_3_Sb^2+^ for the functionalization of inorganic metal–oxide octa­hedra with the generation of doubly bridged M(μ-O)_2_Sb motifs. The latter are formally similar to 1,2-benzene­diolate chelates, which have been observed in mol­ecular organo­anti­mony compounds (Hall & Sowerby, 1980 ▸). Such double bridges are well suited for covalent immobilization of triorgano­anti­mony moieties at the developed metal–oxide surfaces of polyoxometalates. The coordination behaviour of such systems, however, does not appear to have been considered so far. In this context, we have examined a structurally simple and attractive inorganic oxoanion, namely octa­hedral hexa­oxidotellurate(VI). In the present contribution, we crystallize this unit with Ph_3_Sb^2+^ units and report the crystal structure of the title compound, (C_18_H_15_Sb)_3_TeO_6_, which features the formation of discrete clusters, [Te{(μ-O)_2_SbPh_3_}_3_]. 
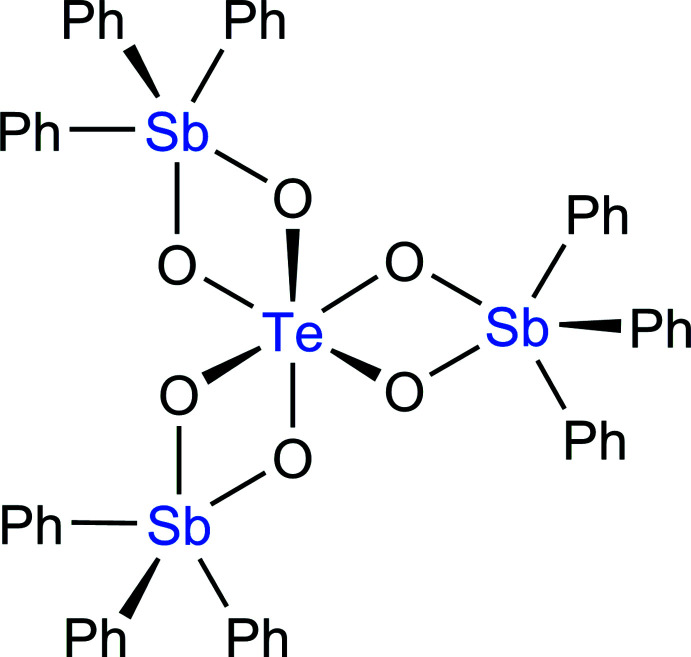


## Structural commentary

**Figure 1:**
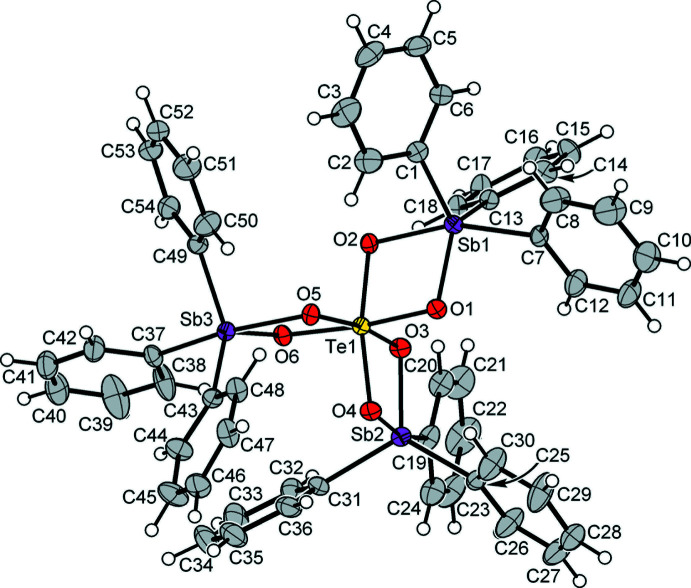
The mol­ecular structure of the title compound with displacement ellipsoids drawn at the 40% probability level. Hydrogen atoms are represented by small circles of arbitrary radius.

The title compound crystallizes in the monoclinic space group, *C*2/*c*, and contains the discrete mol­ecular unit shown in Fig. 1 ▸. The asymmetric tetra­nuclear mol­ecule comprises a [TeO_6_] octa­hedron and three [Ph_3_SbO_2_] polyhedra sharing oxide edges. Thus two oxide bridges are formed from Te^VI^ to each of the three Sb^V^ ions with Te—O—Sb angles in the range 99.33 (13)–102.41 (13)° (Table 1 ▸). The three Te(μ-O)_2_Sb rhombuses are nearly planar, with the maximum deviation of the Te atom from the corresponding mean plane being 0.0676 (12) Å, which occurs in the Te1(μ-O)_2_Sb2 unit. Such fully substituted organometallic hexa­oxotellurate(VI) units are exceedingly rare, with the only known example being an aliphatic Sn^IV^ derivative (Beckmann *et al.*, 2002 ▸). In addition, only two tri­phenyl­tin(IV) analogues of the title compound are known, namely [(Ph_3_SnO)_4_Te(OH)_2_] and [(Ph_3_SnO)_2_Te(OMe)_4_] (Herntrich & Merzweiler, 2010 ▸).

The Te1 atom adopts a slightly distorted octa­hedral coord­ination, with the three *trans* O—Te—O bond angles lying within the range 166.37 (13)–174.49 (13)°. The fivefold coordination around each of three Sb-atoms can best be described as distorted trigonal bipyramidal, with the O2—Sb1—C7 = 161.09 (15)°, O4—Sb2—C19 = 164.73 (16)° and O5—Sb3—C37 = 165.43 (16)° bond angles defining the principal axes of the trigonal bipyramids. This assignment is supported by the calculated five-coordinate τ-indices, which are 0.69, 0.75 and 0.65 for Sb1, Sb2 and Sb3, respectively (Addison *et al.*, 1984 ▸). These values are closer to unity, the value expected for a perfect trigonal–bipyramidal geometry, than to zero, which is expected for a square-based pyramidal geometry.

**Figure 2:**
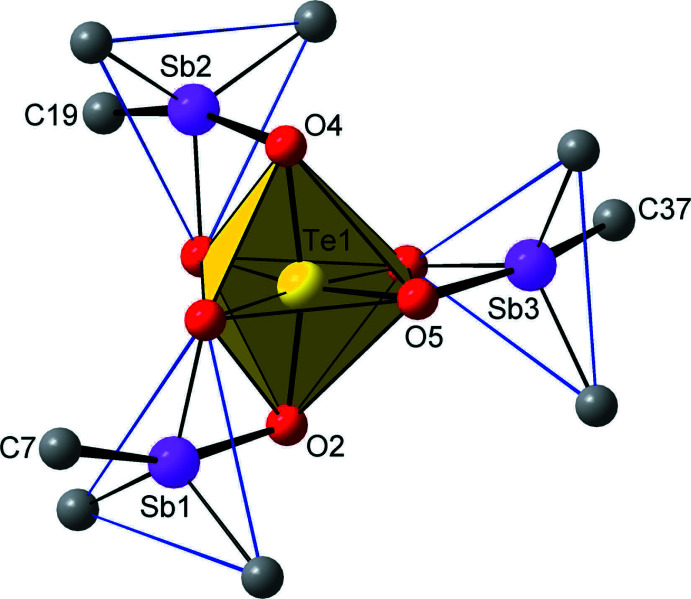
The connection of the Te- and Sb-coordination polyhedra, showing the *mer*-configuration of the [TeO_6_] octa­hedron in the environment of three [SbC_3_O_2_] trigonal bipyramids. The principal axes of the trigonal bipyramids are marked with thick black bonds and their equatorial planes are indicated by blue lines.

In each of the three Sb-based trigonal bipyramids, the axial Sb—O_ax_ bonds, Sb1—O2, Sb2—O4 and Sb3—O5, are slightly longer [in the range 2.087 (3)–2.110 (3) Å] than the equatorial Sb—O_eq_ bonds, Sb1—O1, Sb2—O3 and Sb3—O6 [in the range 1.966 (3)–1.992 (3) Å]. This observation coincides with the differentiation of the Te—O bond lengths; three of which, Te1—O2, Te1—O4 and Te1—O5, lie in the range 1.904 (3)–1.918 (3) Å and three, Te1—O1, Te1—O3 and Te1—O6, lie in the range 1.949 (3)–1.968 (3) Å. Thus when considering the six Te—O—Sb bridges, the shorter Sb—O bonds are accompanied by the longer Te—O bonds and *vice versa*. The distribution of the Te—O_ax_Sb and Te—O_eq_Sb bonds indicates that the coordination octa­hedron around the Te atom has the *mer*-configuration (Fig. 2 ▸). This is consistent with the *mer*-octa­hedral geometry adopted in the previously examined tris­ubstituted tellurates, *e.g. mer*-[(Bu_3_SnO)_3_Te(OH)_3_] (Beckmann *et al.*, 2002 ▸).

## Supra­molecular features

The relatively loose packing of the title compound is dominated by weak dispersion forces, with the calculated packing index of 67.5 approaching the lower limit of the 65-75% range expected for organic solids (Dunitz, 1995 ▸). For comparison, the perceptibly denser packing of more symmetrical polyphenyl substituted species, *e.g.* 1,3,5,7-tetra­phenyl­adamantane, supporting a complex framework of aromatic inter­actions, has a packing index of 70.4 (Boldog *et al.*, 2009 ▸). In the absence of stronger bonding, the present supra­molecular array is mediated by a series of C—H⋯O and C—H⋯π hydrogen bonds with a minor contribution from π/π stacking inter­actions.

**Figure 3:**
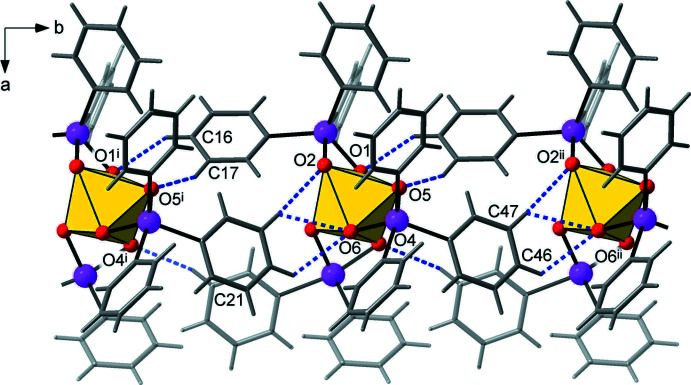
One-dimensional chains running along the *b*-axis direction, in which translation-related mol­ecules of the title compound are linked by a series of weak C—H⋯O hydrogen bonds (shown as dashed blue lines). [Symmetry codes: (i) *x*, *y* − 1, *z*; (ii) *x*, *y* + 1, *z*.]

**Figure 4:**
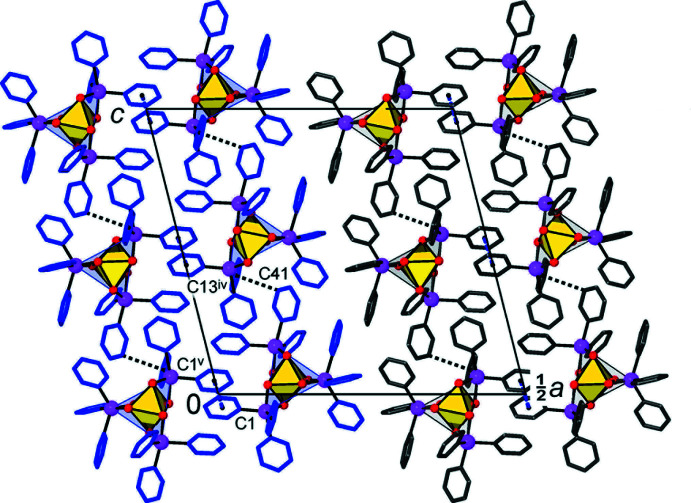
Crystal packing of the title compound, viewed down the *b* axis, showing how the C—H⋯O bonded chains (which are orthogonal to the drawing plane) are connected into layers by means of C–H⋯π and slipped π–π stacking inter­actions. The blue and grey colours indicate two separate bilayers, which lie parallel to the *bc* plane. [Symmetry codes: (iv) *x*, −*y* + 1, *z* + 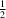 ; (v) −*x*, −*y* + 1, −*z*.]

Very weak mutual C—H⋯O bonding [with the shortest separation C46⋯O6^ii^ = 3.276 (6) Å; symmetry code (ii) *x*, *y* + 1, *z*; Table 2 ▸] arranges the mol­ecules into chains running parallel to the *b* direction (Fig. 3 ▸). Three out of the six above-mentioned inter­actions present are relatively directional, with the angles at the H atoms lying in the range 150-177°. Even weaker C—H⋯π inter­actions are observed between adjacent chains (Fig. 4 ▸). The two shortest of these are C11—H11⋯*Cg*(C43–C48)^iii^ and C41—H41⋯*Cg*(C13–C18)^iv^ (where *Cg* is a ring centroid; symmetry codes: (iii) *x*, −*y* + 2, *x* − 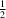 ; (iv) *x*, −*y* + 1, *z* + 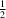 ), with C⋯π separations of 3.775 (6) and 3.505 (6) Å and C—H⋯π angles of 137 and 124°, respectively. This bonding connects the chains into bilayers, which lie parallel to the *bc* plane. In addition, to further consolidate the bilayers, there are weak slipped π–π stacking inter­actions between pairs of inversion-related phenyl rings, with a centroid-to-centroid distance, *Cg*(C1–C6)⋯*Cg*(C1–C6)^v^ = 3.807 (6) Å, an inter­planar distance of 3.603 (5) Å and a slippage angle of 18.8 (5)° [symmetry code: (v) −*x*, −*y* + 1, −*z*]. There are no specific inter­actions between the bilayers, and the shortest of their C⋯C contacts [3.404 (6) Å] is not accompanied by any π–π overlap.

## Database Survey

In the Cambridge Structure Database (CSD, version 5.42, last update November 2020; Groom *et al.*, 2016 ▸), no organo­anti­mony tellurates have been deposited, while only five hits are found for other kinds of organometallic TeO_6_-containing compounds. These include the already mentioned organotin derivatives *trans*-[(Ph_3_SnO)_4_Te(OH)_2_] and *trans*-[(Ph_3_SnO)_2_Te(OMe)_4_] (refcodes: LUWHUH and LUWJAP, Herntrich & Merzweiler, 2010 ▸), *trans*-(Bu_3_SnO)_2_[CH_2_(Ph_2_SnO)_2_]_2_Te (refcode: MOGDER, Beckmann *et al.*, 2002 ▸) and two sil­yloxy compounds bis­(μ_2_-oxo)-octa­kis­(tri­methyl­sil­yloxy)ditellurium and orthotelluric acid tris­(1,1,2,2-tetra­methyl­disilane-1,2-di­yl)ester (refcodes: FAQVUO and FAQWAV, Driess *et al.*, 1999 ▸). The sixth known structure, (Bu_3_SnO)_3_Te(OH)_3_, (Beckmann *et al.*, 2002 ▸) is not deposited in the CSD. All of the above compounds feature sixfold O_6_ octa­hedral coordination of the Te atoms, with just one example of a condensed ditellurate core in (*R*O)_4_Te(μ-O)_2_Te(O*R*)_4_ (*R* = Me_3_Si; FAQVUO). The latter contains double Te—O—Te bridges, which are formally similar to the double Te—O—Sb bridges found in the title compound. No tetra­hedral TeO_4_ fragments have been reported in organometallic series to date. The only known example of a tetra­hedral tellurate is the ionic salt [NEt_4_]_2_TeO_4_·2H_2_O (Konaka *et al.*, 2008 ▸).

## Hirshfeld analysis

**Figure 5:**
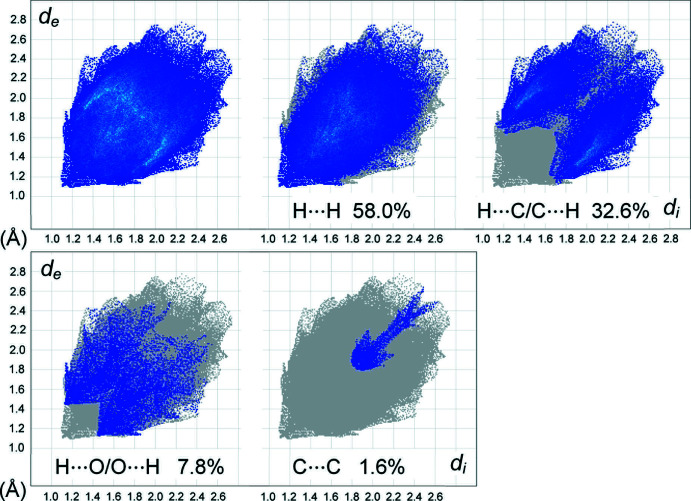
The overall two-dimensional fingerprint plot for the title compound, and those delineated into H⋯H (58.0%), H⋯C/C⋯H (32.6%), H⋯O/O⋯H (7.8%) and C⋯C (1.6%) contacts.

Supra­molecular inter­actions in the title structure were further accessed and visualized by Hirshfeld surface analysis (Spackman & Byrom, 1997 ▸; McKinnon *et al.*, 2004 ▸; Hirshfeld, 1977 ▸; Spackman & McKinnon, 2002 ▸) performed using *CrystalExplorer17* (Turner *et al.*, 2017 ▸). The two-dimensional fingerprint plots (Fig. 5 ▸) suggest that the major contributors to the Hirshfeld surface are H⋯H (58.0%) and H⋯C/C⋯H (32.6%) contacts, while the H⋯O/O⋯H contacts contribute only 7.8%. The latter are identified by a pair of short and very diffuse spikes, at *ca* 2.6 Å, which are actually superimposed upon the regions for the H⋯C/C⋯H inter­actions (the shortest of which is *ca* 2.9 Å). These results are consistent with the weakness of the C—H⋯O bonds in the structure. It is evident that only a few of the H⋯C/C⋯H contacts correspond to C—H⋯π bonding. Therefore, the H⋯C/C⋯H plot represents a rather diffuse collection of points between the pair of poorly resolved features and there no ‘wings’ at the upper left and lower right, which are characteristic of C—H⋯π inter­actions (Spackman & McKinnon, 2002 ▸). The fraction of C⋯C contacts is particularly low (1.6%), indicating only very minor significance of the stacking inter­actions. In fact, with the exception of the one π–π stack noted above, this kind of inter­action is irrelevant to the title structure.

## Synthesis and crystallization

In previously reported syntheses, a range of silver salts were used in ion-exchange reactions to form Ph_3_SbCl_2_ (Goel *et al.*, 1969 ▸) and Ph_4_SbBr (Goel, 1969 ▸) derivatives cleanly and in high yields. Our attempts to prepare tellurate(VI) analogues of such compounds led to de­aryl­ation and the formation of mixtures. The title compound was prepared in low yield by reacting the silver salt, Ag_3_H_3_TeO_6_, with tetra­phenyl­anti­mony(V) bromide as follows:

The starting material, Ag_3_H_3_TeO_6_, was synthesized according to the method of Gospodinov (1992 ▸). 0.220 g (0.4 mmol) of Ag_3_H_3_TeO_6_ were added to a solution containing 0.612 g (1.2 mmol) of Ph_4_SbBr in 20 mL of aceto­nitrile. The mixture was stirred for 3 h and then the AgBr precipitate removed by filtration. Evaporation of the solution yielded a colourless glassy material, which was then dissolved in 10 mL of a 1:1 *v*/*v* mixture of benzene and butyl acetate. Slow evaporation of the solution to a volume of 2–3 mL afforded 0.138 g (27%) of the product in the form of long colourless prisms. The crystals were filtered and dried in air. Analysis (%) for C_54_H_45_O_6_Sb_3_Te: Found: C 50.12, H 3.39; Calculated: C 50.56, H 3.54. IR (KBr, cm^−1^): 454*s*, 520*m*, 610*s*, 692*vs*, 732*vs*, 772*w*, 996*w*, 1066*m*, 1434*s*, 1478*m*, 1576*w*, 2824*w*, 3052*m*.

## Refinement

Crystal data, data collection and structure refinement details are summarized in Table 3 ▸. All the hydrogen atoms were located in difference-Fourier maps and then refined as riding with C—H = 0.95 Å and *U*_iso_(H) = 1.2*U*_eq_(C).

## Supplementary Material

Crystal structure: contains datablock(s) global, I. DOI: 10.1107/S2056989021011294/cq2048sup1.cif

Structure factors: contains datablock(s) I. DOI: 10.1107/S2056989021011294/cq2048Isup2.hkl

CCDC reference: 2118082

Additional supporting information:  crystallographic information; 3D view; checkCIF report

## Figures and Tables

**Table 1:** Selected geometric parameters (Å, °)

Te1—O2	1.904 (3)	Sb2—O3	1.966 (3)
Te1—O5	1.904 (3)	Sb2—O4	2.087 (3)
Te1—O4	1.918 (3)	Sb2—C25	2.136 (5)
Te1—O1	1.949 (3)	Sb2—C31	2.147 (5)
Te1—O6	1.964 (3)	Sb2—C19	2.170 (4)
Te1—O3	1.968 (3)	Sb3—O6	1.967 (3)
Sb1—O1	1.992 (3)	Sb3—O5	2.110 (3)
Sb1—O2	2.091 (3)	Sb3—C49	2.112 (5)
Sb1—C1	2.110 (5)	Sb3—C43	2.122 (4)
Sb1—C13	2.128 (4)	Sb3—C37	2.165 (4)
Sb1—C7	2.175 (4)		
			
O2—Te1—O5	97.67 (13)	O3—Sb2—C25	118.46 (16)
O2—Te1—O4	166.37 (13)	O4—Sb2—C25	88.80 (15)
O5—Te1—O4	94.67 (13)	O3—Sb2—C31	119.67 (17)
O2—Te1—O1	81.53 (12)	O4—Sb2—C31	87.58 (16)
O5—Te1—O1	95.83 (13)	C25—Sb2—C31	118.87 (19)
O4—Te1—O1	91.49 (13)	O3—Sb2—C19	88.62 (17)
O2—Te1—O6	94.01 (13)	O4—Sb2—C19	164.73 (16)
O5—Te1—O6	81.51 (12)	C25—Sb2—C19	101.04 (18)
O4—Te1—O6	93.53 (13)	C31—Sb2—C19	97.6 (2)
O1—Te1—O6	174.49 (13)	O6—Sb3—O5	76.45 (12)
O2—Te1—O3	88.47 (13)	O6—Sb3—C49	116.47 (16)
O5—Te1—O3	168.81 (13)	O5—Sb3—C49	89.13 (16)
O4—Te1—O3	80.35 (12)	O6—Sb3—C43	126.74 (16)
O1—Te1—O3	94.32 (13)	O5—Sb3—C43	87.41 (15)
O6—Te1—O3	88.77 (13)	C49—Sb3—C43	113.58 (18)
O1—Sb1—O2	76.01 (12)	O6—Sb3—C37	89.39 (16)
O1—Sb1—C1	119.50 (15)	O5—Sb3—C37	165.43 (16)
O2—Sb1—C1	91.35 (15)	C49—Sb3—C37	100.46 (19)
O1—Sb1—C13	124.71 (15)	C43—Sb3—C37	98.58 (17)
O2—Sb1—C13	91.45 (14)	Te1—O1—Sb1	102.08 (13)
C1—Sb1—C13	114.34 (17)	Te1—O2—Sb1	100.07 (13)
O1—Sb1—C7	85.22 (15)	Sb2—O3—Te1	102.41 (13)
O2—Sb1—C7	161.09 (15)	Te1—O4—Sb2	99.85 (12)
C1—Sb1—C7	99.99 (18)	Te1—O5—Sb3	99.33 (13)
C13—Sb1—C7	97.49 (17)	Te1—O6—Sb3	102.36 (14)
O3—Sb2—O4	76.38 (12)		

**Table 2:** Hydrogen-bond geometry (Å, °)

*D*—H⋯*A*	*D*—H	H⋯*A*	*D*⋯*A*	*D*—H⋯*A*
C16—H16⋯O1^i^	0.95	2.58	3.342 (6)	137
C17—H17⋯O5^i^	0.95	2.58	3.437 (6)	150
C21—H21⋯O4^i^	0.95	2.75	3.651 (8)	158
C46—H46⋯O6^ii^	0.95	2.67	3.276 (6)	122
C47—H47⋯O6^ii^	0.95	2.73	3.307 (6)	120
C47—H47⋯O2^ii^	0.95	2.70	3.645 (6)	177

Symmetry codes: (i) x, y-1, z; (ii) x, y+1, z.

**Table 3:** Experimental details

Crystal data	
Chemical formula	[Sb_3_Te(C_6_H_5_)_9_O_6_]
*M*_r_	1282.75
Crystal system, space group	Monoclinic, *C*2/*c*
Temperature (K)	173
*a*, *b*, *c* (Å)	47.714 (2), 9.1176 (4), 22.9324 (10)
β (°)	104.168 (4)
*V* (Å^3^)	9672.9 (8)
*Z*	8
Radiation type	Mo *K*α
μ (mm^−1^)	2.31
Crystal size (mm)	0.28 × 0.22 × 0.21

Data collection	
Diffractometer	Stoe IPDS
Absorption correction	Numerical [*X-RED* (Stoe & Cie, 2001 ▸) and *X-SHAPE* (Stoe & Cie, 1999 ▸)]
*T*_min_, *T*_max_	0.499, 0.572
No. of measured, independent and observed [*I* > 2σ(*I*)] reflections	29796, 10754, 8356
*R*_int_	0.053
(sin θ/λ)_max_ (Å^−1^)	0.644

Refinement	
*R*[*F* ^2^ > 2σ(*F* ^2^)], *wR*(*F* ^2^), *S*	0.038, 0.096, 0.93
No. of reflections	10754
No. of parameters	577
H-atom treatment	H-atom parameters constrained
Δρ_max_, Δρ_min_ (e Å^−3^)	1.00, −1.24

Computer programs: *IPDS Software* (Stoe & Cie, 2000 ▸), *SHELXS97* (Sheldrick, 2008 ▸), *SHELXL2018/1* (Sheldrick, 2015 ▸), *DIAMOND* (Brandenburg, 1999 ▸) and *WinGX* (Farrugia, 2012 ▸).

## supplementary crystallographic information

### Crystal data

**Table:**

[Sb_3_Te(C_6_H_5_)_9_O_6_]	*F*(000) = 4976
*M**_r_* = 1282.75	*D*_x_ = 1.762 Mg m^−^^3^
Monoclinic, *C*2/*c*	Mo *K*α radiation, λ = 0.71073 Å
*a* = 47.714 (2) Å	Cell parameters from 8000 reflections
*b* = 9.1176 (4) Å	θ = 2.2–27.2°
*c* = 22.9324 (10) Å	µ = 2.31 mm^−^^1^
β = 104.168 (4)°	*T* = 173 K
*V* = 9672.9 (8) Å^3^	Prism, colorless
*Z* = 8	0.28 × 0.22 × 0.21 mm

### Data collection

**Table:**

Stoe IPDS diffractometer	8356 reflections with *I* > 2σ(*I*)
Radiation source: fine-focus sealed tube	*R*_int_ = 0.053
φ oscillation scans	θ_max_ = 27.2°, θ_min_ = 2.2°
Absorption correction: numerical [X-RED (Stoe & Cie, 2001) and X-SHAPE (Stoe & Cie, 1999)]	*h* = −61→61
*T*_min_ = 0.499, *T*_max_ = 0.572	*k* = −11→11
29796 measured reflections	*l* = −22→29
10754 independent reflections	

### Refinement

**Table:**

Refinement on *F*^2^	Primary atom site location: structure-invariant direct methods
Least-squares matrix: full	Secondary atom site location: difference Fourier map
*R*[*F*^2^ > 2σ(*F*^2^)] = 0.038	Hydrogen site location: inferred from neighbouring sites
*wR*(*F*^2^) = 0.096	H-atom parameters constrained
*S* = 0.93	*w* = 1/[σ^2^(*F*_o_^2^) + (0.0636*P*)^2^] where *P* = (*F*_o_^2^ + 2*F*_c_^2^)/3
10754 reflections	(Δ/σ)_max_ = 0.001
577 parameters	Δρ_max_ = 1.00 e Å^−^^3^
0 restraints	Δρ_min_ = −1.24 e Å^−^^3^

### Special details

**Table:**

Geometry. All esds (except the esd in the dihedral angle between two l.s. planes) are estimated using the full covariance matrix. The cell esds are taken into account individually in the estimation of esds in distances, angles and torsion angles; correlations between esds in cell parameters are only used when they are defined by crystal symmetry. An approximate (isotropic) treatment of cell esds is used for estimating esds involving l.s. planes.

### Fractional atomic coordinates and isotropic or equivalent isotropic displacement parameters (Å^2^)

**Table:**

	*x*	*y*	*z*	*U*_iso_*/*U*_eq_	
Te1	0.11716 (2)	0.68140 (3)	0.04796 (2)	0.02091 (7)	
Sb1	0.07040 (2)	0.57473 (3)	−0.06047 (2)	0.02250 (7)	
Sb2	0.17996 (2)	0.60629 (3)	0.04935 (2)	0.02511 (8)	
Sb3	0.11281 (2)	0.81559 (3)	0.16789 (2)	0.02307 (8)	
O1	0.10263 (7)	0.7188 (3)	−0.03781 (13)	0.0251 (6)	
O2	0.08497 (7)	0.5513 (3)	0.03277 (13)	0.0242 (6)	
O3	0.14157 (7)	0.5152 (3)	0.03645 (14)	0.0272 (7)	
O4	0.15241 (7)	0.7840 (3)	0.04941 (14)	0.0259 (6)	
O5	0.09873 (7)	0.8455 (3)	0.07399 (13)	0.0242 (6)	
O6	0.12791 (7)	0.6419 (3)	0.13479 (13)	0.0256 (6)	
C1	0.02873 (10)	0.6340 (5)	−0.0526 (2)	0.0283 (9)	
C2	0.02482 (13)	0.7519 (6)	−0.0164 (3)	0.0481 (14)	
H2	0.040997	0.806533	0.005257	0.058*	
C3	−0.00268 (15)	0.7882 (7)	−0.0124 (3)	0.0578 (17)	
H3	−0.005373	0.870350	0.011127	0.069*	
C4	−0.02669 (13)	0.7065 (7)	−0.0423 (3)	0.0523 (16)	
H4	−0.045521	0.731029	−0.038595	0.063*	
C5	−0.02249 (12)	0.5895 (7)	−0.0773 (3)	0.0491 (14)	
H5	−0.038624	0.533456	−0.098183	0.059*	
C6	0.00518 (11)	0.5525 (6)	−0.0826 (2)	0.0392 (12)	
H6	0.007804	0.471373	−0.106653	0.047*	
C7	0.06772 (10)	0.6452 (5)	−0.15228 (19)	0.0279 (9)	
C8	0.04299 (14)	0.7081 (8)	−0.1870 (3)	0.0572 (17)	
H8	0.026270	0.718276	−0.171824	0.069*	
C9	0.04280 (16)	0.7567 (10)	−0.2448 (3)	0.075 (2)	
H9	0.025760	0.799698	−0.269074	0.090*	
C10	0.06683 (17)	0.7434 (9)	−0.2672 (3)	0.065 (2)	
H10	0.066485	0.775560	−0.306753	0.078*	
C11	0.09166 (15)	0.6821 (7)	−0.2310 (3)	0.0541 (16)	
H11	0.108536	0.673533	−0.245738	0.065*	
C12	0.09213 (13)	0.6338 (7)	−0.1741 (2)	0.0453 (13)	
H12	0.109299	0.592438	−0.149739	0.054*	
C13	0.07579 (10)	0.3464 (5)	−0.0738 (2)	0.0253 (9)	
C14	0.06436 (11)	0.2885 (5)	−0.1306 (2)	0.0332 (11)	
H14	0.055396	0.351604	−0.162692	0.040*	
C15	0.06591 (12)	0.1384 (5)	−0.1409 (2)	0.0381 (12)	
H15	0.057839	0.098566	−0.179658	0.046*	
C16	0.07947 (13)	0.0479 (5)	−0.0935 (3)	0.0418 (13)	
H16	0.080622	−0.054577	−0.100017	0.050*	
C17	0.09124 (13)	0.1054 (5)	−0.0373 (2)	0.0392 (12)	
H17	0.100771	0.042865	−0.005505	0.047*	
C18	0.08920 (12)	0.2546 (5)	−0.0271 (2)	0.0351 (11)	
H18	0.097003	0.293822	0.011831	0.042*	
C19	0.19842 (11)	0.3885 (5)	0.0503 (2)	0.0339 (11)	
C20	0.17987 (13)	0.2677 (5)	0.0376 (2)	0.0398 (12)	
H20	0.159550	0.282526	0.028564	0.048*	
C21	0.19088 (16)	0.1249 (7)	0.0382 (3)	0.0596 (18)	
H21	0.178264	0.042909	0.029335	0.071*	
C22	0.22051 (19)	0.1063 (7)	0.0519 (4)	0.074 (2)	
H22	0.228237	0.010132	0.052558	0.089*	
C23	0.23914 (15)	0.2243 (8)	0.0646 (3)	0.0610 (19)	
H23	0.259449	0.209139	0.073495	0.073*	
C24	0.22796 (13)	0.3664 (7)	0.0644 (3)	0.0454 (13)	
H24	0.240714	0.447647	0.074062	0.054*	
C25	0.19205 (10)	0.7018 (5)	−0.0260 (2)	0.0296 (10)	
C26	0.21692 (14)	0.6580 (8)	−0.0415 (3)	0.0564 (17)	
H26	0.228689	0.583115	−0.019206	0.068*	
C27	0.22468 (14)	0.7237 (9)	−0.0897 (3)	0.0628 (19)	
H27	0.242017	0.694453	−0.099821	0.075*	
C28	0.20786 (15)	0.8293 (7)	−0.1229 (3)	0.0537 (16)	
H28	0.213614	0.875525	−0.155249	0.064*	
C29	0.18223 (19)	0.8685 (9)	−0.1089 (3)	0.073 (2)	
H29	0.169940	0.939138	−0.132870	0.088*	
C30	0.17436 (15)	0.8053 (7)	−0.0601 (3)	0.0571 (17)	
H30	0.156880	0.833491	−0.050383	0.068*	
C31	0.20271 (10)	0.6711 (6)	0.1384 (2)	0.0324 (10)	
C32	0.21543 (12)	0.5654 (7)	0.1800 (2)	0.0436 (13)	
H32	0.215506	0.465302	0.168523	0.052*	
C33	0.22812 (15)	0.6083 (9)	0.2390 (3)	0.0637 (19)	
H33	0.237072	0.537143	0.267874	0.076*	
C34	0.22770 (15)	0.7527 (10)	0.2555 (3)	0.071 (2)	
H34	0.236135	0.780978	0.295823	0.085*	
C35	0.21508 (13)	0.8571 (8)	0.2136 (3)	0.0560 (16)	
H35	0.215294	0.957248	0.225207	0.067*	
C36	0.20219 (12)	0.8174 (6)	0.1554 (3)	0.0425 (13)	
H36	0.193028	0.889105	0.127008	0.051*	
C37	0.13421 (11)	0.7432 (5)	0.2576 (2)	0.0302 (10)	
C38	0.15596 (16)	0.6418 (7)	0.2671 (3)	0.0572 (18)	
H38	0.162176	0.601976	0.234131	0.069*	
C39	0.1691 (2)	0.5966 (8)	0.3264 (3)	0.079 (3)	
H39	0.183498	0.522309	0.332976	0.095*	
C40	0.16135 (16)	0.6588 (8)	0.3746 (3)	0.0568 (17)	
H40	0.170279	0.628873	0.414496	0.068*	
C41	0.14045 (14)	0.7650 (7)	0.3639 (2)	0.0484 (14)	
H41	0.135249	0.810505	0.397014	0.058*	
C42	0.12681 (13)	0.8076 (6)	0.3065 (2)	0.0428 (13)	
H42	0.112290	0.881184	0.300325	0.051*	
C43	0.12967 (10)	1.0318 (5)	0.1727 (2)	0.0273 (9)	
C44	0.15430 (12)	1.0674 (5)	0.2163 (3)	0.0393 (12)	
H44	0.163334	0.996960	0.245419	0.047*	
C45	0.16574 (14)	1.2099 (6)	0.2169 (3)	0.0506 (15)	
H45	0.183074	1.235275	0.245520	0.061*	
C46	0.15185 (13)	1.3118 (6)	0.1761 (3)	0.0442 (13)	
H46	0.160002	1.406821	0.176095	0.053*	
C47	0.12629 (14)	1.2793 (5)	0.1352 (2)	0.0421 (13)	
H47	0.116082	1.353112	0.109215	0.050*	
C48	0.11567 (12)	1.1371 (5)	0.1323 (2)	0.0341 (11)	
H48	0.098727	1.111817	0.102554	0.041*	
C49	0.06918 (10)	0.8064 (5)	0.1725 (2)	0.0313 (10)	
C50	0.04935 (12)	0.9103 (6)	0.1435 (3)	0.0455 (13)	
H50	0.055228	0.985429	0.120348	0.055*	
C51	0.02094 (13)	0.9043 (8)	0.1483 (3)	0.0571 (17)	
H51	0.007406	0.975992	0.128787	0.069*	
C52	0.01240 (14)	0.7942 (9)	0.1813 (3)	0.0604 (19)	
H52	−0.006883	0.791890	0.185752	0.072*	
C53	0.03177 (14)	0.6876 (8)	0.2080 (3)	0.0540 (17)	
H53	0.025482	0.608218	0.228399	0.065*	
C54	0.06031 (13)	0.6957 (6)	0.2051 (2)	0.0416 (12)	
H54	0.073856	0.625189	0.225552	0.050*	

### Atomic displacement parameters (Å^2^)

**Table:**

	*U*^11^	*U*^22^	*U*^33^	*U*^12^	*U*^13^	*U*^23^
Te1	0.02428 (14)	0.01463 (12)	0.02401 (14)	0.00024 (9)	0.00627 (11)	0.00027 (9)
Sb1	0.02552 (14)	0.01608 (13)	0.02517 (15)	0.00021 (10)	0.00482 (11)	0.00089 (10)
Sb2	0.02550 (15)	0.02110 (14)	0.02942 (16)	0.00203 (10)	0.00803 (12)	0.00078 (11)
Sb3	0.02859 (15)	0.01667 (13)	0.02517 (15)	−0.00063 (10)	0.00892 (11)	0.00024 (10)
O1	0.0306 (16)	0.0175 (13)	0.0268 (15)	−0.0019 (12)	0.0061 (12)	0.0028 (12)
O2	0.0329 (16)	0.0194 (14)	0.0204 (14)	−0.0021 (12)	0.0070 (12)	0.0023 (11)
O3	0.0292 (16)	0.0177 (14)	0.0353 (17)	0.0009 (12)	0.0088 (13)	−0.0024 (12)
O4	0.0295 (16)	0.0148 (13)	0.0338 (17)	−0.0025 (12)	0.0084 (13)	−0.0003 (12)
O5	0.0320 (16)	0.0166 (13)	0.0258 (15)	0.0045 (12)	0.0104 (13)	−0.0015 (11)
O6	0.0323 (17)	0.0184 (14)	0.0259 (15)	0.0011 (12)	0.0068 (13)	−0.0033 (12)
C1	0.030 (2)	0.023 (2)	0.032 (2)	0.0038 (18)	0.0075 (19)	0.0069 (18)
C2	0.042 (3)	0.036 (3)	0.069 (4)	0.005 (2)	0.019 (3)	−0.013 (3)
C3	0.054 (4)	0.049 (3)	0.078 (5)	0.017 (3)	0.031 (3)	−0.002 (3)
C4	0.037 (3)	0.065 (4)	0.061 (4)	0.015 (3)	0.023 (3)	0.020 (3)
C5	0.029 (3)	0.068 (4)	0.050 (3)	−0.006 (3)	0.009 (2)	0.008 (3)
C6	0.032 (3)	0.047 (3)	0.038 (3)	−0.006 (2)	0.008 (2)	−0.003 (2)
C7	0.037 (3)	0.026 (2)	0.020 (2)	−0.0048 (18)	0.0071 (18)	0.0057 (17)
C8	0.045 (3)	0.075 (4)	0.048 (3)	0.000 (3)	0.002 (3)	0.030 (3)
C9	0.056 (4)	0.108 (6)	0.051 (4)	−0.005 (4)	−0.003 (3)	0.042 (4)
C10	0.078 (5)	0.076 (5)	0.039 (3)	−0.011 (4)	0.010 (3)	0.023 (3)
C11	0.063 (4)	0.064 (4)	0.043 (3)	−0.006 (3)	0.028 (3)	0.007 (3)
C12	0.049 (3)	0.055 (3)	0.034 (3)	0.007 (3)	0.015 (2)	0.010 (3)
C13	0.030 (2)	0.0191 (19)	0.027 (2)	0.0001 (16)	0.0080 (18)	−0.0004 (17)
C14	0.035 (3)	0.023 (2)	0.039 (3)	0.0023 (19)	0.003 (2)	−0.0012 (19)
C15	0.049 (3)	0.025 (2)	0.037 (3)	−0.001 (2)	0.003 (2)	−0.006 (2)
C16	0.060 (3)	0.017 (2)	0.052 (3)	0.001 (2)	0.020 (3)	−0.003 (2)
C17	0.064 (4)	0.023 (2)	0.031 (3)	0.011 (2)	0.014 (2)	0.0077 (19)
C18	0.050 (3)	0.026 (2)	0.029 (2)	0.006 (2)	0.011 (2)	0.0035 (19)
C19	0.043 (3)	0.025 (2)	0.039 (3)	0.014 (2)	0.020 (2)	0.0072 (19)
C20	0.050 (3)	0.026 (2)	0.047 (3)	0.011 (2)	0.020 (2)	0.006 (2)
C21	0.077 (5)	0.028 (3)	0.081 (5)	0.020 (3)	0.032 (4)	0.008 (3)
C22	0.099 (6)	0.036 (3)	0.102 (6)	0.032 (4)	0.053 (5)	0.025 (4)
C23	0.055 (4)	0.066 (4)	0.072 (4)	0.037 (3)	0.035 (3)	0.022 (4)
C24	0.045 (3)	0.047 (3)	0.048 (3)	0.019 (3)	0.016 (3)	0.006 (3)
C25	0.031 (2)	0.029 (2)	0.031 (2)	−0.0031 (18)	0.0125 (19)	−0.0010 (18)
C26	0.046 (3)	0.075 (4)	0.057 (4)	0.023 (3)	0.030 (3)	0.027 (3)
C27	0.042 (3)	0.097 (5)	0.056 (4)	0.010 (3)	0.024 (3)	0.025 (4)
C28	0.058 (4)	0.064 (4)	0.046 (3)	−0.010 (3)	0.026 (3)	0.007 (3)
C29	0.095 (6)	0.082 (5)	0.054 (4)	0.036 (5)	0.043 (4)	0.034 (4)
C30	0.063 (4)	0.059 (4)	0.058 (4)	0.025 (3)	0.032 (3)	0.024 (3)
C31	0.023 (2)	0.043 (3)	0.028 (2)	−0.0029 (19)	0.0010 (18)	−0.004 (2)
C32	0.041 (3)	0.053 (3)	0.034 (3)	0.010 (2)	0.003 (2)	0.004 (2)
C33	0.058 (4)	0.091 (5)	0.034 (3)	0.020 (4)	−0.005 (3)	0.005 (3)
C34	0.051 (4)	0.105 (6)	0.045 (4)	0.005 (4)	−0.009 (3)	−0.027 (4)
C35	0.039 (3)	0.066 (4)	0.057 (4)	−0.004 (3)	0.000 (3)	−0.023 (3)
C36	0.033 (3)	0.042 (3)	0.051 (3)	−0.006 (2)	0.007 (2)	−0.012 (2)
C37	0.039 (3)	0.027 (2)	0.022 (2)	−0.0041 (19)	0.0024 (19)	0.0011 (17)
C38	0.080 (5)	0.051 (3)	0.029 (3)	0.032 (3)	−0.007 (3)	−0.005 (2)
C39	0.113 (7)	0.065 (5)	0.046 (4)	0.048 (5)	−0.009 (4)	0.000 (3)
C40	0.077 (5)	0.057 (4)	0.032 (3)	0.004 (3)	0.005 (3)	0.010 (3)
C41	0.056 (4)	0.059 (4)	0.030 (3)	0.002 (3)	0.010 (2)	0.000 (3)
C42	0.046 (3)	0.051 (3)	0.033 (3)	0.005 (3)	0.012 (2)	0.000 (2)
C43	0.033 (2)	0.019 (2)	0.032 (2)	−0.0050 (17)	0.0105 (19)	−0.0021 (17)
C44	0.040 (3)	0.025 (2)	0.048 (3)	−0.004 (2)	0.002 (2)	0.003 (2)
C45	0.052 (3)	0.034 (3)	0.056 (4)	−0.013 (2)	−0.006 (3)	0.002 (3)
C46	0.057 (4)	0.029 (3)	0.046 (3)	−0.015 (2)	0.011 (3)	−0.001 (2)
C47	0.071 (4)	0.023 (2)	0.031 (3)	−0.007 (2)	0.010 (2)	0.0050 (19)
C48	0.049 (3)	0.021 (2)	0.032 (2)	−0.001 (2)	0.009 (2)	0.0022 (18)
C49	0.029 (2)	0.032 (2)	0.034 (2)	−0.0050 (18)	0.0103 (19)	−0.0072 (19)
C50	0.040 (3)	0.045 (3)	0.055 (3)	0.009 (2)	0.017 (3)	0.008 (3)
C51	0.037 (3)	0.072 (4)	0.063 (4)	0.010 (3)	0.013 (3)	−0.010 (3)
C52	0.038 (3)	0.103 (6)	0.043 (3)	−0.024 (4)	0.016 (3)	−0.023 (4)
C53	0.046 (3)	0.079 (5)	0.039 (3)	−0.028 (3)	0.013 (3)	−0.001 (3)
C54	0.045 (3)	0.048 (3)	0.033 (3)	−0.009 (2)	0.010 (2)	0.003 (2)

### Geometric parameters (Å, º)

**Table:**

Te1—O2	1.904 (3)	C22—C23	1.380 (11)
Te1—O5	1.904 (3)	C22—H22	0.9500
Te1—O4	1.918 (3)	C23—C24	1.401 (8)
Te1—O1	1.949 (3)	C23—H23	0.9500
Te1—O6	1.964 (3)	C24—H24	0.9500
Te1—O3	1.968 (3)	C25—C30	1.376 (7)
Sb1—O1	1.992 (3)	C25—C26	1.378 (8)
Sb1—O2	2.091 (3)	C26—C27	1.386 (9)
Sb1—C1	2.110 (5)	C26—H26	0.9500
Sb1—C13	2.128 (4)	C27—C28	1.361 (9)
Sb1—C7	2.175 (4)	C27—H27	0.9500
Sb2—O3	1.966 (3)	C28—C29	1.385 (10)
Sb2—O4	2.087 (3)	C28—H28	0.9500
Sb2—C25	2.136 (5)	C29—C30	1.390 (9)
Sb2—C31	2.147 (5)	C29—H29	0.9500
Sb2—C19	2.170 (4)	C30—H30	0.9500
Sb3—O6	1.967 (3)	C31—C32	1.387 (7)
Sb3—O5	2.110 (3)	C31—C36	1.392 (7)
Sb3—C49	2.112 (5)	C32—C33	1.397 (8)
Sb3—C43	2.122 (4)	C32—H32	0.9500
Sb3—C37	2.165 (4)	C33—C34	1.371 (11)
C1—C6	1.381 (7)	C33—H33	0.9500
C1—C2	1.399 (7)	C34—C35	1.381 (11)
C2—C3	1.378 (8)	C34—H34	0.9500
C2—H2	0.9500	C35—C36	1.376 (8)
C3—C4	1.398 (10)	C35—H35	0.9500
C3—H3	0.9500	C36—H36	0.9500
C4—C5	1.379 (9)	C37—C38	1.367 (8)
C4—H4	0.9500	C37—C42	1.385 (7)
C5—C6	1.396 (8)	C38—C39	1.412 (8)
C5—H5	0.9500	C38—H38	0.9500
C6—H6	0.9500	C39—C40	1.371 (10)
C7—C8	1.377 (7)	C39—H39	0.9500
C7—C12	1.380 (8)	C40—C41	1.368 (9)
C8—C9	1.396 (9)	C40—H40	0.9500
C8—H8	0.9500	C41—C42	1.374 (8)
C9—C10	1.371 (11)	C41—H41	0.9500
C9—H9	0.9500	C42—H42	0.9500
C10—C11	1.387 (10)	C43—C44	1.383 (7)
C10—H10	0.9500	C43—C48	1.387 (7)
C11—C12	1.371 (8)	C44—C45	1.408 (7)
C11—H11	0.9500	C44—H44	0.9500
C12—H12	0.9500	C45—C46	1.368 (8)
C13—C18	1.386 (6)	C45—H45	0.9500
C13—C14	1.387 (7)	C46—C47	1.377 (8)
C14—C15	1.394 (7)	C46—H46	0.9500
C14—H14	0.9500	C47—C48	1.387 (7)
C15—C16	1.391 (7)	C47—H47	0.9500
C15—H15	0.9500	C48—H48	0.9500
C16—C17	1.377 (8)	C49—C54	1.382 (7)
C16—H16	0.9500	C49—C50	1.388 (7)
C17—C18	1.388 (7)	C50—C51	1.387 (8)
C17—H17	0.9500	C50—H50	0.9500
C18—H18	0.9500	C51—C52	1.379 (10)
C19—C24	1.382 (8)	C51—H51	0.9500
C19—C20	1.398 (8)	C52—C53	1.377 (10)
C20—C21	1.403 (7)	C52—H52	0.9500
C20—H20	0.9500	C53—C54	1.382 (8)
C21—C22	1.381 (11)	C53—H53	0.9500
C21—H21	0.9500	C54—H54	0.9500
			
O2—Te1—O5	97.67 (13)	C20—C19—Sb2	118.9 (4)
O2—Te1—O4	166.37 (13)	C19—C20—C21	120.8 (6)
O5—Te1—O4	94.67 (13)	C19—C20—H20	119.6
O2—Te1—O1	81.53 (12)	C21—C20—H20	119.6
O5—Te1—O1	95.83 (13)	C22—C21—C20	118.4 (7)
O4—Te1—O1	91.49 (13)	C22—C21—H21	120.8
O2—Te1—O6	94.01 (13)	C20—C21—H21	120.8
O5—Te1—O6	81.51 (12)	C23—C22—C21	121.5 (6)
O4—Te1—O6	93.53 (13)	C23—C22—H22	119.2
O1—Te1—O6	174.49 (13)	C21—C22—H22	119.2
O2—Te1—O3	88.47 (13)	C22—C23—C24	119.7 (6)
O5—Te1—O3	168.81 (13)	C22—C23—H23	120.2
O4—Te1—O3	80.35 (12)	C24—C23—H23	120.2
O1—Te1—O3	94.32 (13)	C19—C24—C23	120.1 (6)
O6—Te1—O3	88.77 (13)	C19—C24—H24	119.9
O1—Sb1—O2	76.01 (12)	C23—C24—H24	119.9
O1—Sb1—C1	119.50 (15)	C30—C25—C26	119.9 (5)
O2—Sb1—C1	91.35 (15)	C30—C25—Sb2	119.3 (4)
O1—Sb1—C13	124.71 (15)	C26—C25—Sb2	120.7 (4)
O2—Sb1—C13	91.45 (14)	C25—C26—C27	119.7 (6)
C1—Sb1—C13	114.34 (17)	C25—C26—H26	120.1
O1—Sb1—C7	85.22 (15)	C27—C26—H26	120.1
O2—Sb1—C7	161.09 (15)	C28—C27—C26	121.1 (6)
C1—Sb1—C7	99.99 (18)	C28—C27—H27	119.5
C13—Sb1—C7	97.49 (17)	C26—C27—H27	119.5
O3—Sb2—O4	76.38 (12)	C27—C28—C29	119.1 (6)
O3—Sb2—C25	118.46 (16)	C27—C28—H28	120.4
O4—Sb2—C25	88.80 (15)	C29—C28—H28	120.4
O3—Sb2—C31	119.67 (17)	C28—C29—C30	120.5 (6)
O4—Sb2—C31	87.58 (16)	C28—C29—H29	119.7
C25—Sb2—C31	118.87 (19)	C30—C29—H29	119.7
O3—Sb2—C19	88.62 (17)	C25—C30—C29	119.6 (6)
O4—Sb2—C19	164.73 (16)	C25—C30—H30	120.2
C25—Sb2—C19	101.04 (18)	C29—C30—H30	120.2
C31—Sb2—C19	97.6 (2)	C32—C31—C36	120.5 (5)
O6—Sb3—O5	76.45 (12)	C32—C31—Sb2	119.8 (4)
O6—Sb3—C49	116.47 (16)	C36—C31—Sb2	119.4 (4)
O5—Sb3—C49	89.13 (16)	C31—C32—C33	119.0 (6)
O6—Sb3—C43	126.74 (16)	C31—C32—H32	120.5
O5—Sb3—C43	87.41 (15)	C33—C32—H32	120.5
C49—Sb3—C43	113.58 (18)	C34—C33—C32	120.2 (6)
O6—Sb3—C37	89.39 (16)	C34—C33—H33	119.9
O5—Sb3—C37	165.43 (16)	C32—C33—H33	119.9
C49—Sb3—C37	100.46 (19)	C33—C34—C35	120.3 (6)
C43—Sb3—C37	98.58 (17)	C33—C34—H34	119.9
Te1—O1—Sb1	102.08 (13)	C35—C34—H34	119.9
Te1—O2—Sb1	100.07 (13)	C36—C35—C34	120.5 (6)
Sb2—O3—Te1	102.41 (13)	C36—C35—H35	119.7
Te1—O4—Sb2	99.85 (12)	C34—C35—H35	119.7
Te1—O5—Sb3	99.33 (13)	C35—C36—C31	119.4 (6)
Te1—O6—Sb3	102.36 (14)	C35—C36—H36	120.3
C6—C1—C2	119.8 (5)	C31—C36—H36	120.3
C6—C1—Sb1	119.3 (4)	C38—C37—C42	119.4 (5)
C2—C1—Sb1	120.8 (4)	C38—C37—Sb3	121.4 (4)
C3—C2—C1	119.4 (6)	C42—C37—Sb3	119.1 (4)
C3—C2—H2	120.3	C37—C38—C39	119.5 (6)
C1—C2—H2	120.3	C37—C38—H38	120.3
C2—C3—C4	121.2 (6)	C39—C38—H38	120.3
C2—C3—H3	119.4	C40—C39—C38	120.8 (6)
C4—C3—H3	119.4	C40—C39—H39	119.6
C5—C4—C3	118.7 (6)	C38—C39—H39	119.6
C5—C4—H4	120.7	C41—C40—C39	118.5 (5)
C3—C4—H4	120.7	C41—C40—H40	120.8
C4—C5—C6	120.8 (5)	C39—C40—H40	120.8
C4—C5—H5	119.6	C40—C41—C42	121.7 (6)
C6—C5—H5	119.6	C40—C41—H41	119.2
C1—C6—C5	119.9 (5)	C42—C41—H41	119.2
C1—C6—H6	120.0	C41—C42—C37	120.0 (5)
C5—C6—H6	120.0	C41—C42—H42	120.0
C8—C7—C12	120.1 (5)	C37—C42—H42	120.0
C8—C7—Sb1	121.5 (4)	C44—C43—C48	120.4 (4)
C12—C7—Sb1	118.3 (4)	C44—C43—Sb3	120.0 (3)
C7—C8—C9	119.2 (6)	C48—C43—Sb3	119.6 (3)
C7—C8—H8	120.4	C43—C44—C45	118.8 (5)
C9—C8—H8	120.4	C43—C44—H44	120.6
C10—C9—C8	121.0 (6)	C45—C44—H44	120.6
C10—C9—H9	119.5	C46—C45—C44	119.9 (5)
C8—C9—H9	119.5	C46—C45—H45	120.1
C9—C10—C11	118.8 (6)	C44—C45—H45	120.1
C9—C10—H10	120.6	C45—C46—C47	121.3 (5)
C11—C10—H10	120.6	C45—C46—H46	119.3
C12—C11—C10	120.8 (6)	C47—C46—H46	119.3
C12—C11—H11	119.6	C46—C47—C48	119.0 (5)
C10—C11—H11	119.6	C46—C47—H47	120.5
C11—C12—C7	120.2 (5)	C48—C47—H47	120.5
C11—C12—H12	119.9	C43—C48—C47	120.3 (5)
C7—C12—H12	119.9	C43—C48—H48	119.8
C18—C13—C14	119.7 (4)	C47—C48—H48	119.8
C18—C13—Sb1	122.0 (3)	C54—C49—C50	119.6 (5)
C14—C13—Sb1	118.3 (3)	C54—C49—Sb3	119.5 (4)
C13—C14—C15	120.6 (4)	C50—C49—Sb3	120.9 (4)
C13—C14—H14	119.7	C51—C50—C49	120.0 (6)
C15—C14—H14	119.7	C51—C50—H50	120.0
C16—C15—C14	118.9 (5)	C49—C50—H50	120.0
C16—C15—H15	120.5	C52—C51—C50	119.9 (6)
C14—C15—H15	120.5	C52—C51—H51	120.1
C17—C16—C15	120.7 (4)	C50—C51—H51	120.1
C17—C16—H16	119.7	C53—C52—C51	120.1 (6)
C15—C16—H16	119.7	C53—C52—H52	120.0
C16—C17—C18	120.1 (5)	C51—C52—H52	120.0
C16—C17—H17	120.0	C52—C53—C54	120.3 (6)
C18—C17—H17	120.0	C52—C53—H53	119.9
C13—C18—C17	120.0 (5)	C54—C53—H53	119.9
C13—C18—H18	120.0	C53—C54—C49	120.0 (6)
C17—C18—H18	120.0	C53—C54—H54	120.0
C24—C19—C20	119.4 (5)	C49—C54—H54	120.0
C24—C19—Sb2	121.6 (4)		
			
C6—C1—C2—C3	−1.9 (9)	C27—C28—C29—C30	2.7 (12)
Sb1—C1—C2—C3	179.8 (5)	C26—C25—C30—C29	−2.1 (10)
C1—C2—C3—C4	2.1 (10)	Sb2—C25—C30—C29	179.6 (6)
C2—C3—C4—C5	−1.4 (10)	C28—C29—C30—C25	−0.8 (12)
C3—C4—C5—C6	0.5 (9)	C36—C31—C32—C33	−1.0 (9)
C2—C1—C6—C5	1.1 (8)	Sb2—C31—C32—C33	−174.9 (5)
Sb1—C1—C6—C5	179.4 (4)	C31—C32—C33—C34	0.6 (10)
C4—C5—C6—C1	−0.3 (9)	C32—C33—C34—C35	−0.9 (12)
C12—C7—C8—C9	1.5 (10)	C33—C34—C35—C36	1.5 (11)
Sb1—C7—C8—C9	177.5 (6)	C34—C35—C36—C31	−1.9 (10)
C7—C8—C9—C10	−0.4 (12)	C32—C31—C36—C35	1.6 (9)
C8—C9—C10—C11	−0.8 (13)	Sb2—C31—C36—C35	175.5 (5)
C9—C10—C11—C12	0.9 (11)	C42—C37—C38—C39	4.5 (10)
C10—C11—C12—C7	0.3 (10)	Sb3—C37—C38—C39	−179.4 (6)
C8—C7—C12—C11	−1.5 (9)	C37—C38—C39—C40	−3.4 (13)
Sb1—C7—C12—C11	−177.5 (5)	C38—C39—C40—C41	0.4 (13)
C18—C13—C14—C15	0.9 (8)	C39—C40—C41—C42	1.5 (11)
Sb1—C13—C14—C15	−176.5 (4)	C40—C41—C42—C37	−0.4 (10)
C13—C14—C15—C16	−0.9 (8)	C38—C37—C42—C41	−2.7 (9)
C14—C15—C16—C17	−0.1 (9)	Sb3—C37—C42—C41	−178.9 (5)
C15—C16—C17—C18	1.2 (9)	C48—C43—C44—C45	−3.4 (8)
C14—C13—C18—C17	0.2 (8)	Sb3—C43—C44—C45	177.3 (5)
Sb1—C13—C18—C17	177.5 (4)	C43—C44—C45—C46	2.5 (10)
C16—C17—C18—C13	−1.2 (9)	C44—C45—C46—C47	1.7 (10)
C24—C19—C20—C21	−1.1 (8)	C45—C46—C47—C48	−5.0 (10)
Sb2—C19—C20—C21	−179.5 (5)	C44—C43—C48—C47	0.2 (8)
C19—C20—C21—C22	0.4 (10)	Sb3—C43—C48—C47	179.5 (4)
C20—C21—C22—C23	−0.2 (12)	C46—C47—C48—C43	4.0 (9)
C21—C22—C23—C24	0.7 (12)	C54—C49—C50—C51	1.3 (9)
C20—C19—C24—C23	1.5 (9)	Sb3—C49—C50—C51	−178.4 (5)
Sb2—C19—C24—C23	180.0 (4)	C49—C50—C51—C52	−0.7 (10)
C22—C23—C24—C19	−1.4 (10)	C50—C51—C52—C53	−2.1 (10)
C30—C25—C26—C27	3.1 (10)	C51—C52—C53—C54	4.3 (9)
Sb2—C25—C26—C27	−178.6 (6)	C52—C53—C54—C49	−3.7 (9)
C25—C26—C27—C28	−1.2 (12)	C50—C49—C54—C53	0.9 (8)
C26—C27—C28—C29	−1.7 (12)	Sb3—C49—C54—C53	−179.3 (4)

### Hydrogen-bond geometry (Å, º)

**Table:**

*D*—H···*A*	*D*—H	H···*A*	*D*···*A*	*D*—H···*A*
C16—H16···O1^i^	0.95	2.58	3.342 (6)	137
C17—H17···O5^i^	0.95	2.58	3.437 (6)	150
C21—H21···O4^i^	0.95	2.75	3.651 (8)	158
C46—H46···O6^ii^	0.95	2.67	3.276 (6)	122
C47—H47···O6^ii^	0.95	2.73	3.307 (6)	120
C47—H47···O2^ii^	0.95	2.70	3.645 (6)	177

Symmetry codes: (i) *x*, *y*−1, *z*; (ii) *x*, *y*+1, *z*.
